# Supplementary material for: Social support, social context and nonadherence to treatment in young senior patients with multimorbidity and polypharmacy followed-up in primary care. MULTIPAP Study
Source: PLoS One. 2020 Jun 24;15(6):e0235148. doi: 10.1371/journal.pone.0235148 (PMC7314051; doi:10.1371/journal.pone.0235148)
Supplement: Attachment — Submitted filename: Response to Reviewers.docx [file pone.0235148.s001.docx]

**Response to Reviewers:**

**Reviewer #1: The present study addresses a very important topic of social context and nonadherence to treatment in older community dwelling patient with multimorbidity and polypharmacy.**

**The paper is logically structured, methodologically correctly performed and well-written.

I would recommend it for publication subject the following minor revisions.**

1. **The term elderly should be avoided throughout the manuscript and replaced by older people/patients (depending on the context).**

Following the reviewer's indications, we have revised the different terms and the term "elderly" has been replaced by "young seniors". This term was proposed, for the 65-75-year-old population, during the review process of another MULTIPAP study project in the Journal of Medical Internet Research (JMIR).

*Rogero-Blanco E, Lopez-Rodriguez JA, Sanz-Cuesta T, Aza-Pascual-Salcedo M. Bujalance-Zafra MJ, Cura-Gonzalez I; MultiPAP Group. Use of an Electronic Clinical Decision Support System in Primary Care to Assess Inappropriate Polypharmacy* ***in Young Seniors*** *with Multimorbidity: Observational, Descriptive, Cross-Sectional Study. JMIR Med Inform. 2020 Mar 3;8(3):e14130. doi:10.2196/14130. PubMed PMID: 32149715.*

2. **The authors should explain somewhat more in detail why the patients with a life-expectancy less than** **12 months have been excluded.**

This research is nested in the MULTIPAP STUDY, a 12-month RCT cluster that evaluates a complex nonpharmacological intervention in young seniors. One of the exclusion criteria to participate in the trial was a life expectancy of less than 12 months, as the outcome variable (prescription) would be measured at 12 months. We have incorporated this information in the Materials and Methods section.

*Prados-Torres A, del Cura-González I, Prados-Torres D, López-Rodríguez JA, Leiva-Fernández F, Calderón-Larrañaga A, et al. Effectiveness of an intervention for improving drug prescription in primary care patients with multimorbidity and polypharmacy: study protocol of a cluster randomized clinical trial (Multi-PAP project). Implement Sci. 2017;12: 54. doi:10.1186/s13012-017-0584-x*

3. **Where there any differences between adherent and nonadherent patients with regard to underlying multimorbidity and related polypharmacy?**

Table 1 compares the baseline characteristics between adherent and non-adherent patients and presents the results for the number of medicines and the number of diseases. No significant differences were found between adherence/nonadherence and the two clinical variables. Following the reviewer#1 recommendation, we have added an additional paragraph about this matter in the results section.

4. **In the discussion section, which otherwise has been fluently written and provides interesting insights I would recommend starting with the strengths of the study and the added value in comparison to existing evidence followed by limitations.**

   The discussion has been rewritten based on reviewer#1 recommendation.

**Reviewer #2: The paper aims to estimate the prevalence of nonadherence to treatment and its relationship with social support in patients with multimorbidity and polypharmacy, which is of major interest since the prevalence of multimorbidity is rising. The manuscript is well written; however, I think the paper would benefit from providing the reader with more information and further adaptations.**

Thank the reviewer for the contributions to improve the quality of the article. In relation to the grammatical aspects, an exhaustive linguistic revision of the text has been carried out, which has been translated by American Journal Experts. We hope that in the current version it will be better understood.

1. **General comment: please provide line numbering.**

We have provided line numbering.

2. **“Population aging has led multimorbidity…”: population aging only? And what about population** **growth?**

In demography, population aging refers to a change in the age structure of the population, where mature and long-lived age groups become larger. There is an increase in the median age resulting from lower fertility rates and higher life expectancy. This demographic change occurs without any associated growth of the population because population aging is also characterized by the maintenance of or a decline in the size of younger age groups. This population aging is a consequence of increased life expectancy, thanks to the success of public health policies and socioeconomic development, resulting in the abundance of a mature and long-lived population and a greater presence of multimorbidity in this population.

*Organización Mundial de la Salud (OMS). Informe mundial sobre el envejecimiento y la salud. J Chem Inf Model. 2015;53: 1689–1699. doi:10.1017/CBO9781107415324.004*

3. **Pay attention to language: “a very common situation”**

We have replaced “a very common situation” by “becoming increasingly prevalent”.

4. **Cut-off for multimorbidity: three or more is also recommended in the elderly.**

We agree with the reviewer to use three or more diseases as a multimorbidity cut-off point in our study. This aspect is covered in the introduction. Although there is no single operational definition of multimorbidity, most studies establish the cut-off point from two diseases. But another study proposes the cut-off point in three diseases, as this threshold is more specific regarding to the identification of patients with complex health needs in both older and young seniors.

5. **“It affects 81.5%...”: please clarify what you mean by “it”.**

We have replaced “it” by “multimorbidity” on the text.

6. **The structure of the introduction could be improved. There are too many separate paragraphs while they can be embedded into each other in order to create more consistency. Currently, the information is fragmented. The transitions are also not clear.**

The introduction has been restructured according to the instructions of the reviewer.

7. **“Underutilization of necessary treatments”. Why? Please explain.**

Two situations are considered within the concept of adequacy of prescription drugs. One of them is that the patient is taking more drugs than he needs due to his clinical condition and age; the other situation is the opposite, that medicines that may be necessary are not being used. For example, in a patient with atrial fibrillation who is not being treated with anticoagulants. These situations are routinely explored using the STOPP/START criteria that are widely applied in clinical practice. Our group has recently published information about this clinical situation in the MULTIPAP study.

An alternative way of explaining it has been included in the manuscript in order to make it easy to understand.

*Rogero-Blanco E, Lopez-Rodriguez JA, Sanz-Cuesta T, Aza-Pascual-Salcedo M,Bujalance-Zafra MJ, Cura-Gonzalez I; MultiPAP Group. Use of an Electronic Clinical Decision Support System in Primary Care to Assess Inappropriate Polypharmacy in Young Seniors with Multimorbidity: Observational, Descriptive, Cross-Sectional Study. JMIR Med Inform. 2020 Mar 3;8(3):e14130. doi:10.2196/14130. PubMed PMID: 32149715.*

8. **“****50% of patients with multimorbidity do not comply…”: what are the characteristics of these patients (age, disease status etc.)?**

This information comes from two studies. One of them was carried out on a sample of 202 chronic patients living in Spain. Ages ranged from 21 years to 65 years, with a mean of 56.23 years (SD = 8.98). Among the total sample, 68% were women and 32% were men. Among the participants, 40% had hypertension, 29% had dyslipidemia, 18% had diabetes, and 13% had Chronic Obstructive Pulmonary Disease (COPD). The other study cited is a systematic review of studies conducted in adults and adolescents with different chronic diseases.

Taking into account these considerations, we have modified the description in the manuscript.

9. **“… for patients with cardiovascular disease.”: why do you specifically mention this disease?**

Within the group of patients with multiple chronic diseases, one of the groups in which adherence has been studied in more detail is patients with several cardiovascular diseases. For this reason, reference is made in our work to these diseases in particular. However, if the reviewer considers that it is not appropriate to make such a specific reference, we agree to remove it from the text.

10. **“Components of the social context are the physical environment …”: in which specific manner does the physical environment influence drug adherence? Please explain.**

Several studies have shown how the physical environment in which the individual operates affects his or her lifestyle. For example, the characteristics of the neighborhood in which they live (whether there are green areas nearby, crime, pollution, street cleanliness, among others) affect health-disease determinants. The physical environment in which the individual lives provides barriers and facilitators in terms of health promotion and disease prevention and intervenes in the populations risk behaviors. Therefore, self-care for health, including adherence to treatment, influences the way people live.

11. **Please review the use of commas in the manuscript.**

Punctuation marks have been checked throughout the manuscript.

12. **Clarify the patients within your research question, for example “****in young elderly patients with multimorbidity and polypharmacy”. You could also mention this in the title of the manuscript to be transparent to the reader.**

The term "elderly" has been changed in the title and in the rest of the manuscript to "young seniors". This term was proposed, for the 65-75-year-old population, during the review process of another MULTIPAP study project in the Journal of Medical Internet Research (JMIR).

*Rogero-Blanco E, Lopez-Rodriguez JA, Sanz-Cuesta T, Aza-Pascual-Salcedo M. Bujalance-Zafra MJ, Cura-Gonzalez I; MultiPAP Group. Use of an Electronic Clinical Decision Support System in Primary Care to Assess Inappropriate Polypharmacy* ***in Young Seniors*** *with Multimorbidity: Observational, Descriptive, Cross-Sectional Study. JMIR Med Inform. 2020 Mar 3;8(3):e14130. doi:10.2196/14130. PubMed PMID: 32149715.*

13. **“****followed-up in primary care” ◊ attending primary care**

In Spain, the National Health System has universal coverage, and primary care is the first level of care and the gateway to the health system. In primary care, chronic diseases are followed-up, attended and monitored on an ongoing basis. This is included in the strategies for dealing with chronicity both in the NHS and in the different regions participating in the study, Andalusia, Aragon and Madrid.

*MA LO, A PB. Marco estratégico de la Atención Primaria y Comunitaria. 2019; 37. Available: https://www.redaccionmedica.com/contenido/images/Marco Estratégico Atención Comunitaria y Comunitaria 2019 %281%29.pdf*

14. **“****other factors of the social context”: such as? Please explain these factors in brackets.**

Other factors" is removed and replaced by "social context", which has been defined in the introduction.

**METHODS**

1. **Please clarify the “****analytical approach”.**

The "analytical approach" refers to the fact that is a descriptive cross-sectional observational study completed with an analytical methodology such as multivariate models.

2. **Please provide more information on how multimorbidity was measured: how many diseases where measured, which diseases were involved, explain why you used a cut off value of ≥ 3 diseases to define multimorbidity, self-reported or diagnosed by a GP? Only CHRONIC diseases?**

Multimorbidity is the coexistence of multiple chronic diseases or conditions. The chronic diseases were identified from the patient's electronic medical record. The diagnoses that appeared in the clinical medical record as active chronic diseases were collected and confirmed by the GP. Multimorbidity was defined as having three or more chronic diseases. Although there is no single operational definition of multimorbidity, most studies establish the cut-off point from two, and when one wants to increase its specificity to identify complex health needs and the need to overcome an individual disease approach, the threshold of three or more chronic diseases is used.

These details are integrated into the manuscript.

*Prados-Torres A, Poblador-Plou B, Calderón-Larrañaga A, Gimeno-Feliu LA, González-Rubio F, Poncel-Falcó A, et al. Multimorbidity patterns in primary care: Interactions among chronic diseases using factor analysis. PLoS One. 2012;7. doi: 10.1371/journal.pone.0032190*

3. **Please provide more information about the interview during consultation. What was the methodology of the interview? Was it a structured interview? Did the GP went through the validated questionnaires with the patient? Please explain.**

All data were collected by the GP through an interview during the consultation. All the information was collected in a structured way following a study protocol, for which the GPs were previously trained. An electronic data collection notebook was designed and integrated the variables collected by the study, including the validated questionnaires. Each professional accessed the form from their personal computer through the project website (www.multipap.es) with a personal identification code.

We have integrated more data about the interview in the manuscript.

4. **Please provide more details about the Morisky-Green test. Validity? Items?**

The Morisky-Green test is an indirect method for studying adherence. It is the most widely method for studying adherence used in our environment. It consists in a series of 4 questions with a dichotomous yes/no answer, which reflects the patient's behavior with respect to adherence. The aim is to assess whether the patient adopts correct attitudes in relation to the treatment of his/her illness. Patients are considered adherent if they answer the four questions correctly and nonadherent if they fail three or fewer questions: “*Do you ever forget to take the medicines to treat your illness; Do you take the medicines at the indicated times; When you feel well, do you stop taking the medicine; If you ever feel bad, do you stop taking the medicine?”*

A sentence has been added to the methodology for clarify it

The validation of the Morisky-Green questionnaire in Spain offered a 53 for sensitivity, 63 for specificity, 49 for PPV and 66 for NPV, being the best validated test among the indirect methods of assessing non-compliance with treatment.

*Villafaina Barroso A, Gavilán Moral E, et. al. (2011) ‘Pacientes polimedicados frágiles, un reto para el sistema sanitario’, Inf Ter Sist Nac Salud, 35(4), pp. 114–123.*

5. **You measured structural and functional social support. Why did you not measure the third item of social support, namely the informative item as mentioned in the introduction?**

Our study was conducted using the baseline information of the patients included in the MULTIPAP STUDY. This study was carried out with a quantitative methodology which allows us to study the social structural and functional support. Other methodological approaches are required to study social support information. The most commonly used technique for the study of social support information is the qualitative group interview, from which narrative statements are extracted.

*Gottlieb BH, Bergen AE. Social support concepts and measures. J Psychosom Res. 2010;69: 511–520. doi: 10.1016/j.jpsychores.2009.10.001*

6. **You measured structural support based on marital status and number of cohabitants in the home. Is there a validated instrument for measuring structural support?**

Structural support is defined as the number and pattern of direct and indirect social ties surrounding the individual. To study this type of social support there is no validated instrument. Until now, the different investigations have focused mainly on its study through the closest social relationships as a nuclear family and its members.

*Uchino BN, Cacioppo JT, Kiecolt-Glaser JK. The relationship between social support and physiological processes: A review with emphasis on underlying mechanisms and implications for health. Psychol Bull. 1996;119: 488–531. doi:10.1037/0033-2909.119.3.488*

7. **Retirement status: which categories? How defined?**

Retirement was collected as a dichotomous variable (retired/non-retired). In Spain, retirement refers to the administrative act by which an active worker (whether self-employed or employed) moves to a passive or inactive work situation, by reaching a certain maximum legal working age. This allows the person to leave working life and obtain remuneration for the rest of his life. The retirement age in Spain is between 65-67 years old.

We have collected in the manuscript the possible categories that this variable can have.

8. **CNO-12: please provide more information about this instrument.**

The National Classification of Occupations, proposed by the Spanish Society of Epidemiology (CNO-SEE12), was used, which consists of seven social classes that can be grouped into a smaller number of categories, depending on the needs of the study.

In our study, the following seven categories were initially used:

I. Directors and managers of establishments with 10 or more salaried employees and professionals traditionally associated with university degrees.

II. Directors and managers of establishments with fewer than 10 employees, professionals traditionally associated with university diplomas, other technical support professionals, athletes, and artists.

III. Intermediate occupations: administrative salaried employees and professionals supporting administrative management and other services.

IV. Self-employed workers.

V. Supervisors and workers in skilled technical occupations.

VI. Skilled workers in the primary sector and semiskilled workers.

VII. Unskilled workers.

In the context of this study, it was decided to group this variable, taking into account the frequency of each category, as shown in Table 1.

This clarification has been included in the Materials and Methods section.

*Domingo-Salvany A, Bacigalupe A, Carrasco JM, Espelt A, Ferrando J, Borrell C. Propuestas de clase social neoweberiana y neomarxista a partir de la Clasificación Nacional de Ocupaciones 2011. Gac Sanit. 2013;27: 263–272. doi: 10.1016/j.gaceta.2012.12.009*

9. **It is not clear how you determined socioeconomic status: is it based on educational level? Income? Occupational status? What do you mean by social class and socioeconomic level? Please clarify.**
10. **You use income level as a surrogate for the individual's socioeconomic status? What are the cut-off values for income based on? In my opinion, this does not adequately reflect socioeconomic status. Why did you made this choice? This must be further explained in the limitations.**

Socioeconomic level is an economic and sociological measure that combines a person’s job preparedness and their economic and social position in relation to other people, based on the income, education, and employment. Therefore, we agree that the socioeconomic status is a multidimensional concept that cannot be measured solely based on the monthly income.

In the manuscript, the term “socioeconomic status” among the variables has been replaced by “monthly income”, categorized as follows: ≤550 €; 551 to 800 €; 801 to 1,050 €; 1,051 to 1,300 €; 1,301 to 1,550 €; 1,551 to 1,850 €; 1,851 to 2,250 €; 2,251 to 2,700 €; 2,701 to 3,450 €; and >3,450 €. We started with ≤550 € because this is the minimum pension received in Spain in 2015. These categories were finally grouped into ≥2,251 €/month, 1,051-2,250 €/month, ≤1,050 €/month, and NS/NC.

The study of social context in this study also included variables such as educational level, social class, and physical environment through indicators of subjective urban vulnerability and monthly income of the family unit.

To study social classes, the proposed classification of CNO-SEE12 was chosen, which takes into account the type of work performed and skills. This classification consists of 12 classes, of which three are related to owners (capitalists, small employers, and petty bourgeoisie) and nine are related to salaried employees, taking into account three levels of authority (manager, supervisor, and worker) and three skill levels (expert, semiexpert, and nonexpert).

*Domingo-salvany A, Bacigalupe A, Miguel J, Sociedad D, Carrasco JM, Espelt A, et al. Propuestas de clase social neoweberiana y neomarxista a partir de la Clasificación Nacional de Ocupaciones 2011. Gac Sanit. 2013;27: 263–272. doi:10.1016/j.gaceta.2012.12.009*

11. **EQ5D5L --> EQ-5D-5L.**

We have replaced “EQ5D5L” by “EQ-5D-5L” on all the manuscript.

12. **What are the implications of using the EQ-5D?**
13. **Please provide more information on the EQ-5D-5L: which value set did you use to obtain the utilities? Are you interested in both the utilities and VAS scores? Please explain the difference between both scores.**

Quality of life is widely understood as a multidomain concept, with physical, psychological, and social components. To measure this variable, Patient-Reported Outcome (PRO) instruments are used. A PRO is a measure of any aspect of the patient’s health that comes directly from the patient (that is, without patient responses being interpreted by the interviewer).

EQ-5D-5L is a generic questionnaire adapted to Spanish and validated in our environment. This questionnaire measures the health-related quality of life using PROs and consists of two parts.

The first part (D5) is composed of five questions about the health status of the subject and explores five dimensions: mobility, self-care, usual activities, pain/discomfort, and anxiety/depression. Each dimension is measured on a five-point scale.

Based on these five questions, a single weighted health score is derived, called the utility index. A higher score indicates a better state of health. An algorithm proposed for Spain was used to construct this index.

*Herdman M, Gudex C, Lloyd A, et al. Development and preliminary testing of the new five-level version of EQ-5D (EQ-5D-5L). Qual Life Res 2011 Dec;20(10):1727-36.*

*Van Hout B, Janssen MF, Feng YS, et al. Interim scoring for the EQ-5D-5L: mapping the EQ-5D-5L to EQ-5D-3L value sets. Value Health 2012 Jul;15(5):708-15.*

The second part consists of a visual analog scale (VAS). It is a horizontal fixed line (usually 100 mm), with the ends defined as the extreme limits of the scale, with no words describing the intermediate positions, ranging from 0 (worst state) to 100 (best possible health state). Patients are instructed to place a mark on the line corresponding to their perceived state.

Studying the perspective of a patient through the VAS and utility index provides valuable information that can be filtered through an evaluation that offers results on the health-related quality of life.

*Services, H. (2006) ‘Guidance for industry: Patient-reported outcome measures: Use in medical product development to support labeling claims: Draft guidance’, Health and Quality of Life Outcomes, 4, pp. 1–20. doi: 10.1186/1477-7525-4-79.*

14. **There is a difference between quality of life and health-related quality of life. A clear distinction must be made. Both are used interchangeably in this paper.**

The concept of quality of life is broader than that of state of health, since it also considers social welfare (social support, family, economic situation...). Health-related quality of life consists of assessing the subjective influence of the state of health, health care, and health prevention and promotion activities on the individual's ability to achieve and maintain a level of functioning that makes it possible to achieve vital objectives and is reflected in general well-being. The fundamental dimensions are: physical, psychological-cognitive and social functioning.

We have unified the term throughout the document by focusing on health-related quality of life.

*Shumaker SA, Naughton MJ. The international assessment of health-related quality of life: a*

*theoretical perspective. En: Shumaker SA, Berzon R, eds. Quality of life: theory, translation,*

*measurement & analysis. Oxford: Rapid comunications of Oxford Ltd., 1995: 3-10*

15. **What is the reference number of the approval of the Ethics Committee?**

The project was approved by the Clinical Research Ethics Committee of Aragon (CEICA) on 30 September 2015 with reference number PI15/0217.

The reference number has been included in the manuscript.

**RESULTS + DISCUSSION**

1. **What was the response rate?**

This study was conducted using the information collected at the baseline visit of the patients included in the MULTIPAP Study. Of the 636 patients who were offered to participate, 593 were eventually included. Given the characteristics of the study, all the patients included were interviewed at the baseline visit, so this study presents the data of 100% of the sample.

2. **“errors in self-observation”: please clarify.**

Many authors have pointed out that self-reporting methods have the potential to over-report adherence or fail to disclose non-adherence due to recall bias, lack of data, concerns about social appropriateness, and failures in self-observation.

We have replaced "errors in self-observation" with "failures in self-observation" for better understanding.

*Malmen M, Raval AD, Bennett BM, Borah BJ, Hutchins DS, Manias E, et al. Science Direct Methods for Measuring Multiple Medication Adherence: A Systematic Review e Report of the ISPOR Medication Adherence and Persistence Special Interest Group. 2019;2. doi: 10.1016/j.jval.2018.08.006*

3. **Why do you think specifically these five items of social support are associated with nonadherence?**

The vital moment for a young senior is characterized by the onset of physical and social changes. Some of the physical, mental, and social capabilities of young seniors begin to be affected, and above all, this moment is marked by their leaving the working world. This situation causes a change in how patients perceive themselves and their social relationships. For this reason, it makes sense that for a young senior, certain things, such as a praise and recognition, become important when he or she does something well. It is essential for them to receive useful advice about important events, social invitations, love and affection, and help when sick in bed. As some studies have shown, feeling social support and being understood by others can be a motivation to improve adherence to treatment.

*Cottrell E, Yardley S. Lived experiences of multimorbidity: An interpretative meta-synthesis of patients’, general practitioners’ and trainees’ perceptions. Chronic Illn. 2015;11: 279–303. doi:10.1177/1742395315574764*

4. **“Cuellar-Flores et al”: provide the year of this publication in brackets**

This error has been corrected

5. **“a representative sample of the general population”: please explain.**

In Spain, 90% of the population is treated in primary care health centres. The sample participating in this study has been selected among the general population that goes to the health centres, so we can say that this sample is representative of the general population of Young seniors with multimorbility and polymedication.

We have given another form to the paragraph since after reading it carefully and having incorporated the recommendations of Reviewer 1 we have seen fit to improve it.

6. **Comparison with other studies, first paragraph: and what about the influence of polypharmacy and multimorbidity?**

Unlike other studies, the present study found no significant difference between being adherent and the number of drugs or diseases. We think that this may be due to the fact that among the patients included in the sample (with more than 5 drugs prescribed and more than 3 chronic diseases) there is not much variability in the number of drugs and diseases making it difficult to obtain significant statistical difference.

7. **“the experiences lived by patients in the context of multimorbidity”: what was the age class of these patients?**

The interpretive meta-synthesis study included 25 qualitative studies, of which the most studied age range was 65-85 years, and the widest studied age range was 30-90 years. All of them with multi-morbidity.

A clarification has been made in the manuscript.

*Cottrell E, Yardley S. Lived experiences of multimorbidity: An interpretative meta-synthesis of patients’, general practitioners’ and trainees’ perceptions. Chronic Illn. 2015;11: 279–303. doi:10.1177/1742395315574764*

8. **“… passively cope…”: this is interesting. What could be the explanation why these patients experience a positive effect of incomprehension from their environment?**

People who feel that others do not want to understand their situation experience negative effects in terms of health management, causing them to passively cope with their illness, instead of actively coping.

Multimorbidity and polymedication lead to a situation that entails multiple health appointments and frequent monitoring by health professionals, which is often accompanied by tests, feelings of dependence, and a decreased ability, while the presence of symptoms can affect their daily lives and social relationships. This situation can cause patients to feel social isolation and a lack of understanding.

9. **You often mention qualitative research papers; is there also quantitative evidence?**

Quantitative studies on treatment adherence and social support evaluate overall functional support without offering insights into its components. A strength of our study is that it places a special emphasis on the items of functional social support and its relationship with adherence to treatment, thus further delving into the components of functional support.

10. **HRQoL: you compare the VAS score with those aged 65 years older without information on multimorbidity and polypharmacy, and the VAS scores are almost similar. This is interesting because the patients in the study are patients with multimorbidy and polypharmacy. Are there any explanations for this result?**

The study by Azpiazu Garrido M. et al. did not collect multimorbidity or polymedication data. However, a large part of the population of this age present with these conditions. Therefore, it is not surprising that the populations in the above study and our study are very similar. We think that this similarity is because the study populations represent a young senior age group. This type of patients, despite presenting with multimorbidity and polymedication, are not yet in a situation of dependency or perception of their quality of life as impaired by their health status.

11. **Implications of the study findings: can you give a practical example of how the socioeconomic context/quality of life/preferences of the patient could be taken into account when anticipating towards a more effective approach?**

There are certain situations in which patients encounter barriers to adherence. For example, they cannot afford the prescribed treatment, do not understand the form of administration, or do not have anyone to help with care; in these cases, we should offer them a social resource. It is also possible that the problem that we are treating is not important for the patient because he or she is more concerned about another health issue that affects their perceived quality of life to a greater extent; in this case, it will be more effective to address their priorities first.

The social approach of the patient is essential, and adherence cannot be addressed if social aspects are ignored.

12. **“reflecting aspects linked to both the physical and social environment…”: and what about the mental component?**

In this work, we did not address the mental component, having decided that it requires a special study. A more comprehensive approach is one of the objectives of another study within the MULTIPAP Study.
